# Type of Track and Trigger system and incidence of in-hospital cardiac arrest: an observational registry-based study

**DOI:** 10.1186/s12913-020-05721-5

**Published:** 2020-09-18

**Authors:** Helen Hogan, Andrew Hutchings, Jerome Wulff, Catherine Carver, Elizabeth Holdsworth, Jerry Nolan, John Welch, David Harrison, Nick Black

**Affiliations:** 1 Department of Health Services Research and Policy, London School of Hygiene and Tropical Medicine, Room 117, 15-17 Tavistock Place, London, WC1H 9SH UK; 2 Intensive Care National Audit and Research Centre, Napier House, 24 High Holborn, London, WC1V 6AZ UK; 3 Royal United Hospital Bath NHS Trust, Combe Park, Bath, BA1 3NG UK; 4 Critical Care Outreach, University College London Hospitals NHS Foundation Trust, 235 Euston Rd, Fitzrovia, London, NW1 2BU UK

**Keywords:** Track and Trigger system, National Early Warning Score, Electronic Track and Trigger system, In-hospital cardiac arrest, Deterioration

## Abstract

**Background:**

Failure to recognise and respond to patient deterioration on hospital wards is a common cause of healthcare-related harm. If patients are not rescued and suffer a cardiac arrest as a result then only around 15% will survive. Track and Trigger systems have been introduced into the NHS to improve both identification and response to such patients. This study examines the association between the type of Track & Trigger System (TTS) (National Early Warning Score (NEWS) versus non-NEWS) and the mode of TTS (paper TTS versus electronic TTS) and incidence of in-hospital ward-based cardiac arrests (IHCA) attended by a resuscitation team.

**Methods:**

TTS type and mode was retrospectively collected at hospital level from 106 NHS acute hospitals in England between 2009 to 2015 via an organisational survey. Poisson regression and logistic regression models, adjusted for case-mix, temporal trends and seasonality were used to determine the association between TTS and hospital-level ward-based IHCA and survival rates.

**Results:**

The NEWS was introduced in England in 2012 and by 2015, three-fifths of hospitals had adopted it. One fifth of hospitals had instituted an electronic TTS by 2015. Between 2009 and 2015 the incidence of IHCA fell. Introduction or use of NEWS in a hospital was associated with a reduction of 9.4% in the rate of ward-based IHCA compared to non-NEWS systems (incidence rate ratio 0.906, *p* < 0.001). The use of an electronic TTS was also associated with a reduction of 9.8% in the rate of IHCA compared with paper-based TTS (incidence rate ratio 0.902, *p* = 0.009). There was no change in hospital survival.

**Conclusions:**

The introduction of standardised TTS and electronic TTS have the potential to reduce ward-based IHCA. This is likely to be via a range of mechanisms from early intervention to institution of treatment limits. The lack of association with survival may reflect the complexity of response to triggering of the afferent arm of the rapid response system.

## Background

Resuscitation teams are called to between one and five in-hospital cardiac arrests (IHCA) per 1000 hospital admissions amounting to around 20,000 arrests in NHS hospitals in England each year [1]. Survival to discharge is around 13–20% [2, 3]. These IHCA often reflect a failure to manage antecedent events – case reviews have shown that many patients exhibited signs of deterioration (physiological changes or level of consciousness) for up to 8 h beforehand [4, 5]. Better identification and management of patient deterioration will reduce avoidable mortality and also ensure that patients at the natural end of life are not harmed at this critical time by receipt of inappropriate interventions.

Track and Trigger systems were expected to reduce the incidence of IHCA by identifying deterioration at an earlier stage when there is greater opportunity to intervene and provide timely and appropriate care, be that increased monitoring, clinical review or the revision of decisions around limits of treatment. It is perhaps not surprising that an inconsistent picture of the impact of TTS on IHCA has emerged from studies over the last two decades [6–8].

Despite this uncertainty there has been widespread uptake of a variety of TTS across the NHS [9]. Amid concerns that lack of standardisation might endanger patients [10], the Royal College of Physicians of London introduced the National Early Warning Score (NEWS) in 2012.This was rapidly adopted across England, particularly following evaluations showing it performed at least as well as and often better than, other existing TTS already in place [11]. Additionally, electronic versions of TTSs have been introduced, including for NEWS [12]. These have advantages over paper-based systems by countering known problems through mandating entry of a full set of patient observations, accurately calculating scores and, in some cases, automatically sending an alert to an appropriate responder when a particular score threshold is met [13, 14].

The National Cardiac Arrest Audit (NCAA), a collaboration between the Intensive Care National Audit and Research Centre (ICNARC) and the Resuscitation Council (UK) was started in 2009 with the aim of collecting data on IHCA that elicit a resuscitation team response [15]. The audit currently receives reports from over 80% of hospitals in England, representative of the range of hospitals found in the NHS. The availability of this longitudinal data from a large number of organisations alongside variation in hospitals’ TTS configuration, provides an opportunity to evaluate the impact of implementation of NEWS and electronic TTS on the incidence of ward-based IHCA in England.

## Methods

Full details on definitions, sampling, data sources, model development and analysis are reported elsewhere [16]. Briefly, we carried out an observational study to determine any association between the type of TTS (NEWS versus non-NEWS) and mode of TTS (paper versus electronic) and incidence of ward-based IHCA and survival [17].

Data for organisational interventions were available from a survey of NCAA hospitals conducted in 2015. These data were collected at organisation and not patient level. Survey respondents were asked to provide details of the TTS in place at each hospital for each year and quarter between 2009 and 2015. Of all 171 hospitals participating in the NCAA in 2015, 139 (81.3%) responded, of which 122 provided up to 6 years of historic data on the TTS used across wards from which two primary variables were derived for each year and quarter:

- *Type of TTS-NEWS/non-NEWS:* hospitals were categorised as either using NEWS (which included both original NEWS and NEWS to which a limited number of extra items (most commonly urine output) had been added locally or non-NEWS TTS.
- *Mode of TTS-Electronic/Paper:* hospitals were categorised as either using paper-based or electronic TTS (TTS could be any type, including NEWS)*.*

Hospitals that operated dual systems over the index period, for example, both paper-based and electronic during a transition to electronic TTS, were categorised according to the predominant system in use across the hospital during the relevant quarter.

Hospital-level data on ward-based IHCA over time for each participating hospital was provided by NCAA [18]. Hospitals had to have a minimum of 3 months consecutive audit data to be eligible for inclusion. IHCA in NCAA were linked to mortality data from the Office for National Statistics to identify deaths following discharge from hospital.

Data on all hospital inpatient admissions from Hospital Episode Statistics (HES) was used to derive denominators for estimating ICHA rates and, via linkage with NCAA patients, to enable case-mix adjustment. Patient-level variables used in case mix adjustment included emergency/non-emergency admission, main diagnosis and comorbidity (modified Charlson score) [19]. The proportion of admissions with a main diagnosis of atherosclerotic heart disease was used as a hospital-level variable.

### Analysis

An initial descriptive analysis was undertaken to determine types and mode of TTS over time, characteristics of patients who had an IHCA, and trends in IHCA rates and hospital survival.

A random effects Poisson regression analysis adjusted for case mix, temporal trend and seasonality was conducted. Interventions were initially modelled as either a difference in level or a difference in slopes [20], with the change point based on the quarterly data from the hospital survey response, and incidence rate ratios (IRR) were estimated with 95% confidence intervals. Analyses examined associations between NEWS/non-NEWS and electronic/paper TTS and changes in level or changes in slope for IHCA rates in separate and combined models. A wash-out period of 3 months was used for hospitals that changed from non-NEWS to NEWS TTS or paper to electronic TTS. Analysis was by complete case with the exception of ethnicity where a separate category for missing was included.

Sensitivity analyses examined the impact of the following modifications on the estimated IRRs: no wash-out or 6-month wash-out period; inclusion of all in-hospital IHCA (to include IHCA in non-ward locations); restricting analysis to data from 2011 onwards; and restricting analysis to hospitals that reported a change in type or mode of TTS during the study.

The associations between TTS and overall hospital survival in all admissions were examined using a similar case-mix adjusted model. The associations between TTS and 30-day survival among ward-based IHCA were examined using logistic regression with case-mix adjustment extended to include the length of hospital stay prior to the 2222 call, the presenting or first documented rhythm and the reason for admission to hospital.

### Patient involvement

There were two patient representative members of the study Scientific Advisory Group who contributed to the development of the research questions and oversight of study implementation.

## Results

### Hospital and patient sample

**Fig. 1:**
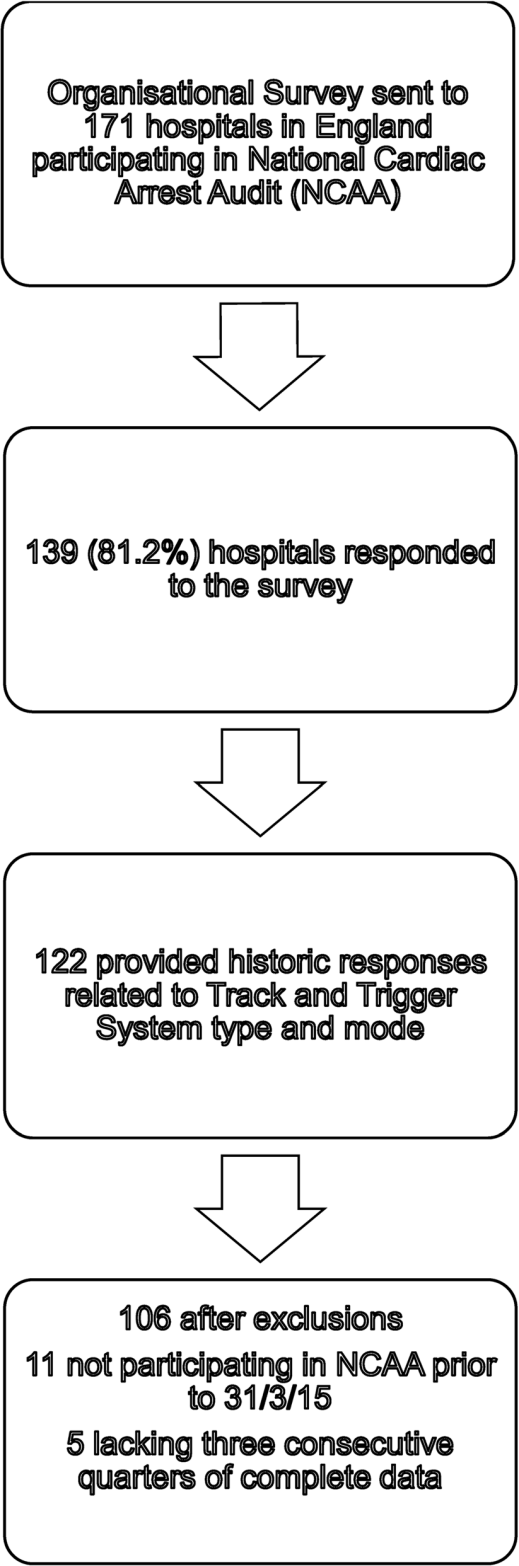
Hospital sampling process

Of the 139 hospitals participating in the NCAA in 2015 that completed the organisational questionnaire, 106 were eligible for the analysis (Fig. 1). Hospitals varied in the duration of their eligibility with a median of 14 consecutive quarters of eligible data (range 3 to 22 quarters). *Supplementary Table* *S1* shows the number of hospitals contributing data for each quarter; the number grew to 104 in 2014 as more hospitals participated in NCAA and declined in the final two quarters because of the impact of the requirement for two quarters of data following any change in a hospital’s TTS provision. The 106 hospitals were representative, based on region, number of admissions and IHCA numbers of all hospitals in NCAA in 2015 (70% of all English acute hospitals) (*Supplementary Table* *S2*). Overall, there were 21,595 patients having had 22,057 IHCA based on 13,059,865 hospital admissions. Compared with all hospital admissions, those who experienced an IHCA were more likely to be male (56.2 v 40.4%), 75 years or older (34.7 v 27.2%), have two or more comorbidities (53.9 v 23.3%) and be an emergency admission (91.8 v 66.0%) (Table 1).

**Table 1:** Characteristics of patients experiencing in-hospital ward-based cardiac arrests and all hospital admissions

	In-hospital cardiac arrests (*n* = 21,595)	All Admissions (*n* = 13,059,865)
**Age category**		
Under 65	35,736,557 (16.6)	7,558,437 (57.9)
65–74	4198 (19.4)	1,953,500 (15.0)
75–84	7496 (34.7)	2,127,250 (16.3)
85 and older	6328 (29.3)	1,420,677 (10.9)
Not stated or missing	0	1
**Sex**		
Male	12,143 (56.2)	5,276,200 (40.4)
Female	9452 (43.8)	7,783,187 (59.6)
Not stated or missing	0	478
**Ethnicity**		
White	18,945 (87.7)	10,922,469 (83.6)
Asian/Asian British	752 (3.5)	620,412 (4.8)
Black/Black British	349 (1.6)	314,316 (2.4)
Any other ethnic group	232 (1.1)	276,563 (2.1)
Not stated or missing	1317 (6.1)	926,105 (7.1)
**Deprivation (Index of Multiple Deprivation by decile)**		
Least deprived 10%	1635 (7.6)	1,048,095 (8.1)
Less deprived 10–20%	1732 (8.0)	1,123,433 (8.7)
Less deprived 20–30%	2036 (9.5)	1,204,167 (9.3)
Less deprived 30–40%	2215 (10.3)	1,282,332 (9.0)
Less deprived 40–50%	2184 (10.1)	1,272,685 (9.8)
More deprived 40–50%	2192 (10.2)	1,263,100 (9.8)
More deprived 30–40%	2337 (10.9)	1,328,799 (10.3)
More deprived 20–30%%	2247 (10.4)	1,360,653 (10.5)
More deprived 10–20%	2246 (10.4)	1,431,102 (11.1)
Most deprived 10%	2705 (12.6)	1,637,906 (12.7)
Missing	66	107,593
**Charlson index of comorbidity**		
No comorbidity	3525 (16.3)	6,636,490 (50.8)
One comorbidity	6426 (29.8)	3,382,501 (25.9)
Two comorbidities	5863 (27.1)	1,811,933 (13.9)
Three or more comorbidities	5781 (26.8)	1,228,941 (9.4)
Missing	0	0
**Admission method**		
Non-Emergency	1777 (8.2)	4,444,978 (34.0)
Emergency	19,813 (91.8)	8,613,205 (66.0)
Missing	5	1682

### Interventions

All 106 hospitals used some form of TTS. The first use of a NEWS TTS was reported in 2012 and by the end of 2014 around 60% of hospitals used a NEWS TTS. There were 52 hospitals that switched from a non-NEWS to a NEWS TTS during the period they were eligible and contributed data for analysis. The remaining hospitals used either a non-NEWS (42 hospitals) or a NEWS (12 hospitals) TTS during their entire period of eligibility.

The majority of hospitals used a paper-based TTS. Use of an electronic TTS was first reported in 2010 and by 2014 around 20% of hospitals used electronic TTS. Eighteen hospitals switched from paper to an electronic system during their period of eligibility, 86 were paper-based and two operated an electronic system throughout the period.

There were 48 hospitals that used the same type and mode of TTS throughout their period of eligibility. Twelve hospitals switched from both non-NEWS to NEWS and paper to electronic TTS during their period of eligibility, four of which made both switches in the same year and quarter.

### Trends in IHCA and outcomes

**Fig. 2:**
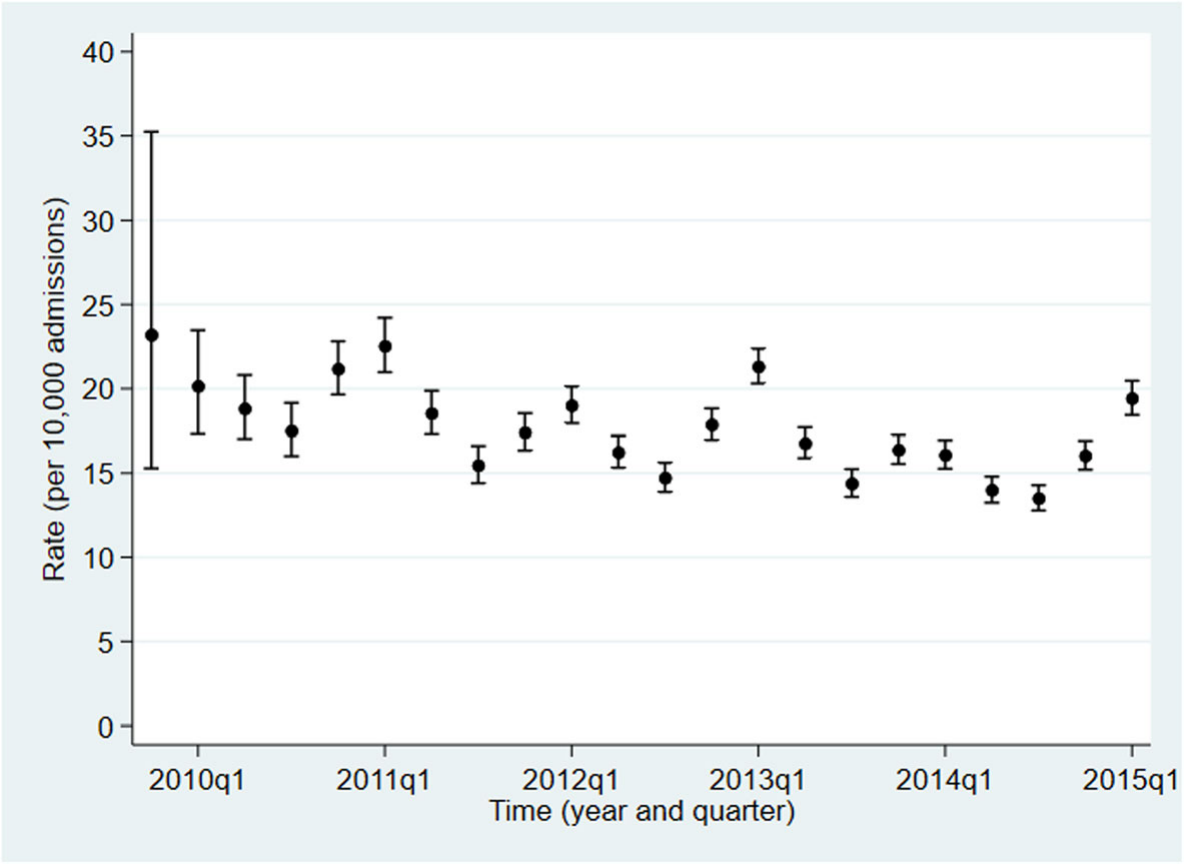
Trend in the crude rate of ward-based in-hospital cardiac arrests attended by the resuscitation team in 13 million hospital admissions

The incidence of IHCA decreased between 2009 and 2015 (Fig. 2). When adjusted for case-mix and seasonality the observed decrease was 7.4% per year.

### Association between interventions and IHCA incidence

Table 2 shows the associations between type and mode of TTS and ward-based IHCA rates in 13 million hospital admissions after adjusting for case-mix, time trends and quarterly seasonality. Differences in levels were retained in the final model for both TTS interventions.

**Table 2:** Associattion between TTS interventions and in-hospital ward-based cardiac arrest rates in 106 NCAA hospitals

Track and trigger	Case mix adjusted incidence rate ratio (95%CI)		
	Individual intervention^a^	Combined slopes and levels^b^	Combined interventions^c^
Non-NEWS	reference	reference	reference
NEWS/NEWS-based	0.892 (0.849, 0.938)	0.871 (0.825, 0.919)	0.906 (0.861, 0.954)
*p*-value for difference in levels	*P* < 0.001	*P* < 0.001	*P* < 0.001
Annual trend: non-NEWS	0.935 (0.923, 0.948)	0.945 (0.932, 0.958)	
Annual trend: NEWS/NEWS-based	0.903 (0.873, 0.935)	0.959 (0.921, 0.998)	
p-value for difference in slopes	*P* = 0.089	*P* = 0.513	
Paper	reference	reference	reference
Electronic	0.878 (0.814, 0.947)	0.883 (0.814, 0.958)	0.902 (0.835, 0.975)
p-value for difference in levels	*P* = 0.001	*P* = 0.003	*P* = 0.009
Annual trend: paper	0.935 (0.924, 0.946)	0.938 (0.927, 0.949)	
Annual trend: electronic	0.864 (0.823, 0.906)	0.894 (0.849, 0.942)	
p-value for difference in slopes	*P* = 0.002	*P* = 0.080	

^a^each TTS intervention modelled separately as either a difference in level or a difference in slopes

^b^each TTS intervention modelled as both difference in level and slopes

^c^both TTS interventions modelled as difference in levels

The use of NEWS was associated with a 9.4% reduction in the rate of IHCA compared with a non-NEWS TTS (IRR 0.906, 95% CI 0.861, 0.954, *p* = 0.001). Use of an electronic TTS was associated with a 9.8% reduction in the rate of IHCA compared with a paper TTS (IRR 0.902, 95% CI 0.835, 0.975, *p* = 0.009). See *Supplementary Table* *S3* for full model results.

Sensitivity analysis showed similar associations between hospital-wide IHCA rates and NEWS TTS (IRRs 0.909 to 0.918) in the different models. Associations with an electronic TTS were more sensitive to alternative specifications (IRRs 0.879 to 0.953). *Supplementary Table* *S4* shows these associations when restricting the hospital sample to only those hospitals that changed type or mode of TTS during the study period. Associations with changes in mode become non-significant.

### Association between interventions and survival

Background trends across the 106 hospitals showed an increase in the rate of hospital survival of 0.21% per year and an increase in the odds of 30-day survival in ward-based IHCA of 5.4% per year. However, unlike IHCA rates, there was no evidence that type or mode of TTS was associated with a difference in survival (Table 3). See *Supplementary Tables* *S5**a* and *S5**b* for full model results.

**Table 3:** Association between TTS interventions and survival in 106 NCAA hospitals

Track and trigger	30-day survival in IHCA	Hospital survival in all admissions
	Case mix adjusted odds rate ratio (95%CI)	Case mix adjusted incidence rate ratio (95%CI)
Non-NEWS	reference	Reference
NEWS	1.105 (0.946, 1.292)	0.9999 (0.9982, 1.0015)
*p*-value for difference in levels	*P* = 0.209	*P* = 0.861
Paper	Reference	Reference
Electronic	1.150 (0.922, 1.435)	1.0010 (0.9987, 1.0034)
*p*-value for difference in levels	*P* = 0.214	*P* = 0.401

## Discussion

### Main findings

Between 2009 and 2015 the incidence of IHCA attended by hospital resuscitation teams fell. All hospitals were using a TTS in 2009, with uptake of NEWS occurring fairly rapidly during 2012. By the end of 2014, 60% of hospitals were using NEWS. When compared with a non-NEWS system, introduction of NEWS was associated with a 9.4% reduction in the rate of ward-based IHCA in addition to the background trend. Similarly, use of an electronic TTS, compared with paper TTS, was also associated with a reduction of 9.8% in the rate of IHCA after controlling for the effect of a NEWS system.

### Explanations of findings

Introduction of NEWS across the NHS has standardised the collection of physiological observations, risk scoring and response criteria and electronic collection would be expected to bring further enhancements by increasing the completeness of recording, reducing miscalculation of scores and in some cases, reducing the time between the patient reaching a threshold score and appropriate response [13, 14]. Our observation that NEWS and electronic TTS are associated with fewer ward-based IHCA may be due to ward staff making earlier interventions [21], improved timeliness of referrals to staff with critical care skills [14, 22], or through initiation of treatment limitation decisions (e.g. use of Do Not Attempt Cardiopulmonary Resuscitation (DNACPR) decisions). We found no association with either overall hospital mortality or survival post-arrest. A lack of association with overall hospital survival may represent a signal to noise issue. The signal from the relatively few deaths that TTSs may prevent not being detectable above the noise of small improvements in survival across all admissions [1]. The association between TTS and post-arrest survival is likely to be complicated given the range of different impacts TTS might have on the risk of survival for those experiencing an arrest.

The association of TTS with IHCA and mortality has been inconsistent in previous studies, although there is clearer evidence that TTS improves the recording of observations [23]. Such inconsistencies may reflect the differences between the interventions being studied, the outcomes measured or the fidelity of the implementation [8, 13, 24–26]. Our study adds weight to the evidence of an association between TTS on IHCA when implemented in a real-world context, but findings related to mortality, indicate that mechanisms associated with this association may be complex.

### Strengths and limitations

Large rigorous observational studies provide an alternative approach to randomised controlled trials when an intervention is already in place. The NCAA provided an objective measure of IHCA in a large number of acute hospitals.

However, only known confounding factors which have been measured and collected can be accounted for in adjustments for case-mix. There may have been some unknown or unmeasured confounders not taken into account. Modelling the hospitals as random effects provided some protection against unmeasured hospital-level confounding factors.

The study relied on the accurate reporting of the TTS interventions and ward-based cardiac arrests in the hospitals. Misspecification in either would generally reduce the study’s ability to detect a any association. The risk of under-reporting of IHCA to NCAA is small as 87% of hospitals reported a case ascertainment via the organisational survey of more than 90%. Furthermore, limiting the focus to type and mode of TTS reduced the risk of statistically significant findings arising by chance but at the same time necessitated simplification of organisational arrangements.

The natural experiment of different hospitals introducing the intervention at different times provides some protection against confounding by secular trends [17]. A difference-in-difference approach was also considered but rejected because there were insufficient control hospitals for matching to hospitals that switched TTS.

Finally, given that staff may respond to rising TTS scores in a range of ways from simple resuscitation measures to consideration of adoption of end of life care pathways, changes across the NHS such as wider use of formal DNACPR decisions are likely to be contributing to reductions in cardiac arrest rates associated with TTS use. DNACPR decisions will remove patients from the pool of deteriorating patients likely to arrest. The overall effect of these changes is difficult to assess due to lack of national trend data on DNACPR [27]. However, there is evidence that rapid response teams (RRT) are playing an increasing role in such decision-making when called to the wards [28, 29]. One international study showed around a third of treatment limitation decisions (including DNACPR) were made after the alerting of a RRT, often in response to TTS criteria [30], and other studies, that between a quarter and a third of RRT encounters result in new treatment limitation decision [31, 32]. This represents a welcome benefit of TTS and should be seen as one of the explanatory mechanisms for the observed association with lower rates of IHCA.

## Conclusions

Standardisation of TTS and the introduction of systems that both facilitate correct score calculation and automate the triggering of a response may lead to a reduction in ward-based IHCA through a range of mechanisms. Further research is required to develop a clearer understanding of this relationship and the response mechanism that is associated with the observed changes. This should be accompanied by the development of a greater understanding of the impact of different elements of electronic NEWS on outcomes and the effect of introducing laboratory results into the scoring system. This would help determine the added value that electronic tools might bring to reducing IHCA compared to paper-based TTS.

## Supplementary information

**Additional file 1 **: **Table S1.** Summary of hospital eligible for inclusion in the analysis by year and quarter.

**Additional file 2 **: **Table S2.** Comparison of the hospitals included in final sample with those excluded and all hospitals in the National Cardiac Arrest Audit.

**Additional file 3 **: **Table S3.** Full model results (presented as incidence rate ratios and 95% confidence intervals) for models of the association between TTS interventions and in-hospital ward-based cardiac arrest rates.

**Additional file 4 **: **Table S4.** Impact of interventions on IHCAs after restriction to hospitals reporting a change in intervention.

**Additional file 5 **: **Table S5a.** Full model results (presented as odds ratios and 95% confidence intervals) for models of the association between TTS interventions and 30-day survival following IHCA. **Table S5b.** Full model results (presented as incidence rate ratios and 95% confidence intervals) for models of the association between TTS interventions and hospital survival in all admissions.

## Data Availability

The datasets generated and analysed during this current study are not publicaly available. This study utilised a linked NCAA-HES-ONS dataset that cannot be disseminated further due to conditions attached to initial release to the authors. All queries should be submitted to the corresponding author.

## Abbreviations

DNACPR: Do Not Attempt Cardiopulmonary Resuscitation
HES: Hospital Episode Statistics
IHCA: In-hospital ward-based cardiac arrests
ICNARC: Intensive Care National Audit and Research Centre
IRR: Incidence rate ratio
NCAA: National Cardiac Arrest Audit
NEWS: National Early Warning Score
NHS: National Health System
ONS: Office for National Statistics
TTS: Track & Trigger system
