# Supplementary material for: Type of Track and Trigger system and incidence of in-hospital cardiac arrest: an observational registry-based study
Source: BMC Health Serv Res. 2020 Sep 18;20:885. doi: 10.1186/s12913-020-05721-5 (PMC7501601; doi:10.1186/s12913-020-05721-5)
Supplement: Supplementary file 1 — Additional file 1 : Table S1. Summary of hospital eligible for inclusion in the analysis by year and quarter. [file 12913_2020_5721_MOESM1_ESM.docx]

**Supplementary Table S1 Summary of hospital eligible for inclusion in the analysis by year and quarter**

| **Year and** | **Hospitals** | **Admissions** | **IHCAs** | **NEWS/NEWS-based TTS** | | **Electronic TTS** | |
| --- | --- | --- | --- | --- | --- | --- | --- |
| **quarter** | **n** | **n** | **n** | **n (%)** | **Switched in quarter: n** | **n (%)** | **Switched in quarter: n** |
| 2009 Q4 | 1 | 9,481 | 36 | 0 (0%) | 0 | 0 (0%) | 0 |
| 2010 Q1 | 9 | 82,358 | 254 | 0 (0%) | 0 | 0 (0%) | 0 |
| 2010 Q2 | 23 | 202,440 | 583 | 0 (0%) | 0 | 0 (0%) | 0 |
| 2010 Q3 | 32 | 271,336 | 728 | 0 (0%) | 0 | 1 (3%) | 0 |
| 2010 Q4 | 37 | 325,873 | 1,063 | 0 (0%) | 0 | 1 (3%) | 0 |
| 2011 Q1 | 38 | 331,471 | 1,096 | 0 (0%) | 0 | 1 (3%) | 0 |
| 2011 Q2 | 51 | 430,351 | 1,216 | 0 (0%) | 0 | 1 (2%) | 0 |
| 2011 Q3 | 58 | 492,752 | 1,218 | 0 (0%) | 0 | 1 (2%) | 0 |
| 2011 Q4 | 65 | 553,195 | 1,509 | 0 (0%) | 0 | 2 (3%) | 0 |
| 2012 Q1 | 73 | 622,925 | 1,842 | 1 (1%) | 1 | 2 (3%) | 0 |
| 2012 Q2 | 82 | 705,838 | 1,825 | 3 (4%) | 1 | 2 (2%) | 0 |
| 2012 Q3 | 86 | 757,205 | 1,756 | 6 (7%) | 2 | 4 (5%) | 2 |
| 2012 Q4 | 91 | 789,059 | 2,235 | 10 (11%) | 3 | 4 (4%) | 0 |
| 2013 Q1 | 92 | 770,012 | 2,463 | 14 (15%) | 4 | 4 (4%) | 0 |
| 2013 Q2 | 90 | 769,769 | 1,989 | 23 (26%) | 9 | 5 (6%) | 1 |
| 2013 Q3 | 94 | 816,517 | 1,841 | 31 (33%) | 5 | 7 (7%) | 2 |
| 2013 Q4 | 98 | 854,728 | 2,151 | 45 (46%) | 12 | 10 (10%) | 3 |
| 2014 Q1 | 100 | 871,304 | 2,198 | 54 (54%) | 9 | 11 (11%) | 1 |
| 2014 Q2 | 104 | 896,977 | 1,999 | 58 (56%) | 2 | 19 (18%) | 8 |
| 2014 Q3 | 104 | 919,861 | 1,979 | 62 (60%) | 4 | 20 (19%) | 1 |
| 2014 Q4 | 96 | 858,094 | 2,119 | 58 (60%) | 0 | 19 (20%) | 0 |
| 2015 Q1 | 87 | 728,319 | 2,102 | 52 (60%) | 0 | 19 (22%) | 0 |
| Total |  | 13,059,865 | 34,202 |  | 52 |  | 18 |
