# Supplementary material for: Type of Track and Trigger system and incidence of in-hospital cardiac arrest: an observational registry-based study
Source: BMC Health Serv Res. 2020 Sep 18;20:885. doi: 10.1186/s12913-020-05721-5 (PMC7501601; doi:10.1186/s12913-020-05721-5)
Supplement: Supplementary file 2 — Additional file 2 : Table S2. Comparison of the hospitals included in final sample with those excluded and all hospitals in the National Cardiac Arrest Audit. [file 12913_2020_5721_MOESM2_ESM.doc]

***Supplementary Table S2*** Comparison of the hospitals included in final sample with those excluded and all hospitals in the National Cardiac Arrest Audit

| **Characteristic** | **Included hospitals**  **(N=106)** | **Excluded hospitals**  **(N=65)** | **All hospitals**  **(N=171)** |
| --- | --- | --- | --- |
| **Region** |  |  |  |
| East Midlands | 9 (8.5) | 4 (6.2) | 13 (7.6) |
| East of England | 12 (11.3) | 5 (7.7) | 17 (9.9) |
| London | 16 (15.1) | 14 (21.5) | 30 (17.5) |
| North East | 8 (7.5) | 5 (7.7) | 13 (7.6) |
| North West | 15 (14.2) | 9 (13.8) | 24 (14.0) |
| South Central | 8 (7.5) | 1 (1.5) | 9 (5.3) |
| South East Coast | 11 (10.4) | 3 (4.6) | 14 (8.2) |
| South West | 9 (8.5) | 7 (10.8) | 16 (9.4) |
| West Midlands | 11 (10.4) | 6 (9.2) | 17 (9.9) |
| Yorkshire and the Humber | 7 (6.6) | 11 (16.9) | 18 (10.5) |
| **Number of hospital admissions per year** |  |  |  |
| <50,000 | 27 (25.5) | 18 (27.7) | 45 (26.3) |
| 50,000-99,999 | 61 (57.5) | 35 (53.8) | 96 (56.1) |
| 100,000 or more | 18 (17.0) | 12 (18.5) | 30 (17.5) |
| **Number of IHCA per year** |  |  |  |
| <50 | 19 (17.9) | 6 (9.2) | 25 (14.6) |
| 50-99 | 43 (40.6) | 23 (35.4) | 66 (38.6) |
| 100-149 | 27 (25.5) | 24 (36.9) | 51 (29.8) |
| 150 or more | 17 (16.0) | 12 (18.5) | 29 (17.0) |
| **Length of participation in NCAA** |  |  |  |
| < 1 year | 2 (1.9) | 21 (32.3) | 23 (13.5) |
| 1 year | 4 (3.8) | 8 (12.3) | 12 (7.0) |
| 2 years | 7 (6.6) | 9 (13.8) | 16 (9.4) |
| 3 years | 21 (19.8) | 4 (6.2) | 25 (14.6) |
| 4 years | 30 (28.3) | 11 (16.9) | 41 (24.0) |
| 5 or more years | 42 (39.6) | 12 (18.5) | 54 (31.6) |
