# Supplementary material for: Type of Track and Trigger system and incidence of in-hospital cardiac arrest: an observational registry-based study
Source: BMC Health Serv Res. 2020 Sep 18;20:885. doi: 10.1186/s12913-020-05721-5 (PMC7501601; doi:10.1186/s12913-020-05721-5)
Supplement: Supplementary file 3 — Additional file 3 : Table S3. Full model results (presented as incidence rate ratios and 95% confidence intervals) for models of the association between TTS interventions and in-hospital ward-based cardiac arrest rates. [file 12913_2020_5721_MOESM3_ESM.docx]

***Supplementary Table S3 Full model results (presented as incidence rate ratios and 95% confidence intervals) for models of the association between TTS interventions and in-hospital ward-based cardiac arrest rates***

| **Variables** | **Model 1** | **Model 2** | **Model 3** | **Model 4** | **Model 5** | **Model 6** | **Model 7** |
| --- | --- | --- | --- | --- | --- | --- | --- |
| **Fixed effects parameters** |  |  |  |  |  |  |  |
| NEWS/NEWS-based (vs non-NEWS) | 0.892 (0.849, 0.938) | --- | 0.871 (0.825, 0.919) | --- | --- | --- | 0.906 (0.861, 0.954) |
| Annual trend non-NEWS | --- | 0.935 (0.923, 0.948) | 0.945 (0.932, 0.958) | --- | --- | --- | --- |
| Annual trend NEWS/NEWS-based | --- | 0.903 (0.873, 0.935) | 0.959 (0.921, 0.998) | --- | --- | --- | --- |
| Electronic (vs paper) | --- | --- | --- | 0.878 (0.814, 0.947) | --- | 0.883 (0.814, 0.958) | 0.902 (0.835, 0.975) |
| Annual trend paper | --- | --- | --- | --- | 0.935 (0.924, 0.946) | 0.938 (0.927, 0.949) | --- |
| Annual trend electronic | --- | --- | --- | --- | 0.864 (0.823, 0.906) | 0.894 (0.849, 0.942) | --- |
| Age (vs <25 years) |  |  |  |  |  |  |  |
| 25-34 years | 1.86 (1.36, 2.54) | 1.86 (1.36, 2.54) | 1.86 (1.36, 2.54) | 1.88 (1.38, 2.57) | 1.88 (1.38, 2.57) | 1.88 (1.38, 2.57) | 1.87 (1.37, 2.56) |
| 35-44 years | 3.94 (2.97, 5.25) | 3.95 (2.97, 5.25) | 3.95 (2.97, 5.25) | 4.01 (3.02, 5.34) | 4.01 (3.02, 5.34) | 4.01 (3.02, 5.34) | 4.00 (3.00, 5.34) |
| 45-54 years | 7.04 (5.34, 9.26) | 7.04 (5.35, 9.26) | 7.04 (5.35, 9.26) | 7.23 (5.50, 9.52) | 7.23 (5.50, 9.52) | 7.23 (5.50, 9.51) | 7.15 (5.42, 9.43) |
| 55-64 years | 11.4 (8.71, 15.0) | 11.4 (8.71, 15.0) | 11.4 (8.71, 15.0) | 11.7 (8.89, 15.3) | 11.7 (8.89, 15.3) | 11.7 (8.89, 15.3) | 11.6 (8.79, 15.2) |
| 65-74 years | 16.6 (12.7, 21.8) | 16.6 (12.7, 21.8) | 16.6 (12.7, 21.8) | 16.9 (12.9, 22.1) | 16.9 (12.9, 22.1) | 16.9 (12.9, 22.1) | 16.8 (12.8, 22.1) |
| 75-84 years | 23.8 (18.2, 31.1) | 23.8 (18.2, 31.1) | 23.8 (18.2, 31.1) | 24.4 (18.6, 31.9) | 24.4 (18.6, 31.9) | 24.4 (18.6, 31.9) | 24.1 (18.4, 31.6) |
| 85+ years | 28.7 (21.9, 37.5) | 28.7 (21.9, 37.5) | 28.7 (21.9, 37.5) | 29.3 (22.4, 38.4) | 29.3 (22.4, 38.4) | 29.3 (22.4, 38.4) | 29.0 (22.1, 38.1) |
| Female (vs male) | 0.736 (0.716, 0.756) | 0.736 (0.716, 0.756) | 0.736 (0.716, 0.756) | 0.737 (0.717, 0.757) | 0.737 (0.717, 0.757) | 0.737 (0.717, 0.757) | 0.736 (0.716, 0.757) |
| Ethnicity (vs white) |  |  |  |  |  |  |  |
| Asian/Asian British | 1.18 (1.09, 1.27) | 1.18 (1.09, 1.27) | 1.18 (1.09, 1.27) | 1.15 (1.07, 1.25) | 1.15 (1.07, 1.25) | 1.15 (1.07, 1.25) | 1.17 (1.09, 1.27) |
| Black/Black British | 1.07 (0.955, 1.19) | 1.07 (0.954, 1.19) | 1.07 (0.955, 1.19) | 1.04 (0.933, 1.16) | 1.04 (0.933, 1.16) | 1.04 (0.933, 1.16) | 1.05 (0.937, 1.17) |
| Any other ethnic group | 0.903 (0.789, 1.03) | 0.902 (0.788, 1.03) | 0.902 (0.788, 1.03) | 0.925 (0.812, 1.05) | 0.927 (0.813, 1.06) | 0.926 (0.812, 1.06) | 0.905 (0.790, 1.04) |
| Not stated or missing | 1.19 (1.13, 1.26) | 1.19 (1.12, 1.26) | 1.19 (1.13, 1.26) | 1.19 (1.12, 1.26) | 1.18 (1.12, 1.25) | 1.19 (1.12, 1.25) | 1.20 (1.13, 1.27) |
| Decile of Index of Multiple Deprivation (vs least deprived 10%) |  |  |  |  |  |  |  |
| Less deprived 10-20% | 0.985 (0.920, 1.05) | 0.984 (0.919, 1.05) | 0.984 (0.920, 1.05) | 0.989 (0.924, 1.06) | 0.990 (0.925, 1.06) | 0.989 (0.925, 1.06) | 0.983 (0.919, 1.05) |
| Less deprived 20-30% | 1.04 (0.970, 1.11) | 1.03 (0.969, 1.11) | 1.04 (0.969, 1.11) | 1.04 (0.975, 1.11) | 1.04 (0.976, 1.11) | 1.04 (0.976, 1.11) | 1.03 (0.968, 1.11) |
| Less deprived 30-40% | 1.04 (0.977, 1.11) | 1.04 (0.975, 1.11) | 1.04 (0.977, 1.11) | 1.04 (0.974, 1.11) | 1.04 (0.975, 1.11) | 1.04 (0.975, 1.11) | 1.04 (0.976, 1.11) |
| Less deprived 40-50% | 1.04 (0.976, 1.11) | 1.04 (0.973, 1.11) | 1.04 (0.975, 1.11) | 1.04 (0.977, 1.11) | 1.04 (0.978, 1.11) | 1.04 (0.978, 1.11) | 1.04 (0.977, 1.11) |
| More deprived 40-50% | 1.09 (1.02, 1.17) | 1.09 (1.02, 1.16) | 1.09 (1.02, 1.17) | 1.10 (1.03, 1.17) | 1.10 (1.03, 1.17) | 1.10 (1.03, 1.17) | 1.09 (1.02, 1.17) |
| More deprived 30-40% | 1.10 (1.03, 1.18) | 1.10 (1.03, 1.18) | 1.10 (1.03, 1.18) | 1.11 (1.04, 1.18) | 1.11 (1.04, 1.18) | 1.11 (1.04, 1.18) | 1.10 (1.03, 1.18) |
| More deprived 20-30% | 1.07 (1.00, 1.14) | 1.07 (0.997, 1.14) | 1.07 (0.999, 1.14) | 1.07 (1.00, 1.14) | 1.07 (1.00, 1.14) | 1.07 (1.00, 1.14) | 1.07 (0.998, 1.14) |
| More deprived 10-20% | 1.04 (0.975, 1.12) | 1.04 (0.973, 1.11) | 1.04 (0.974, 1.11) | 1.05 (0.981, 1.12) | 1.05 (0.982, 1.12) | 1.05 (0.982, 1.12) | 1.04 (0.972, 1.11) |
| Most deprived 10% | 1.08 (1.01, 1.16) | 1.08 (1.01, 1.15) | 1.08 (1.01, 1.16) | 1.08 (1.01, 1.15) | 1.08 (1.01, 1.15) | 1.08 (1.01, 1.15) | 1.08 (1.01, 1.16) |
| Charlson index of comorbidity (vs no comorbidity) |  |  |  |  |  |  |  |
| One comorbidity | 1.50 (1.43, 1.56) | 1.50 (1.43, 1.56) | 1.50 (1.43, 1.56) | 1.49 (1.43, 1.56) | 1.49 (1.43, 1.55) | 1.49 (1.43, 1.55) | 1.50 (1.43, 1.56) |
| Two comorbidities | 1.82 (1.74, 1.90) | 1.82 (1.74, 1.90) | 1.82 (1.74, 1.90) | 1.82 (1.74, 1.90) | 1.81 (1.73, 1.90) | 1.81 (1.73, 1.90) | 1.82 (1.74, 1.90) |
| Three or more comorbidities | 2.02 (1.92, 2.12) | 2.02 (1.92, 2.12) | 2.02 (1.92, 2.12) | 2.02 (1.92, 2.12) | 2.02 (1.92, 2.12) | 2.02 (1.92, 2.12) | 2.02 (1.92, 2.12) |
| Emergency admission | 2.66 (2.53, 2.80) | 2.66 (2.53, 2.80) | 2.66 (2.53, 2.80) | 2.67 (2.54, 2.81) | 2.68 (2.55, 2.81) | 2.68 (2.55, 2.81) | 2.66 (2.53, 2.80) |
| Cardiac comorbidity | 1.41 (1.36, 1.46) | 1.41 (1.36, 1.46) | 1.41 (1.36, 1.46) | 1.41 (1.36, 1.46) | 1.41 (1.36, 1.46) | 1.41 (1.36, 1.46) | 1.41 (1.37, 1.46) |
| Main diagnosis (vs any other) |  |  |  |  |  |  |  |
| Circulatory | 1.76 (1.70, 1.82) | 1.76 (1.70, 1.82) | 1.76 (1.70, 1.82) | 1.75 (1.69, 1.81) | 1.75 (1.69, 1.81) | 1.75 (1.69, 1.81) | 1.76 (1.70, 1.82) |
| Respiratory | 1.86 (1.79, 1.92) | 1.86 (1.79, 1.92) | 1.86 (1.79, 1.92) | 1.86 (1.80, 1.93) | 1.86 (1.80, 1.93) | 1.86 (1.80, 1.93) | 1.86 (1.79, 1.93) |
| Pregnancy | 0.0415  (0.0172, 0.100) | 0.0415  (0.0172, 0.100) | 0.0415  (0.0172, 0.100) | 0.0409  (0.0169, 0.0989) | 0.0409  (0.0169, 0.0989) | 0.0409  (0.0169, 0.0989) | 0.0420  (0.0174, 0.101) |
| Annual trend | 0.943 (0.930, 0.955) | --- | --- | 0.933 (0.922, 0.944) | --- | --- | 0.946 (0.934, 0.959) |
| Season (vs Jan-Mar) |  |  |  |  |  |  |  |
| Apr-Jun | 0.854 (0.822, 0.887) | 0.852 (0.820, 0.885) | 0.855 (0.823, 0.888) | 0.859 (0.828, 0.892) | 0.861 (0.829, 0.894) | 0.860 (0.828, 0.893) | 0.858 (0.825, 0.891) |
| Jul-Sep | 0.812 (0.781, 0.843) | 0.811 (0.780, 0.842) | 0.812 (0.782, 0.844) | 0.813 (0.783, 0.844) | 0.813 (0.783, 0.844) | 0.813 (0.783, 0.844) | 0.813 (0.782, 0.845) |
| Oct-Dec | 0.911 (0.879, 0.944) | 0.911 (0.878, 0.944) | 0.911 (0.879, 0.945) | 0.913 (0.881, 0.946) | 0.912 (0.880, 0.945) | 0.913 (0.881, 0.946) | 0.910 (0.878, 0.943) |
| **Random effects parameters** |  |  |  |  |  |  |  |
| Alpha | 0.0641  (0.0468, 0.0879) | 0.0785  (0.0575, 0.107) | 0.0665  (0.0484, 0.0913) | 0.0653  (0.0479, 0.0892) | 0.0761  (0.0558, 0.104) | 0.0699  (0.0510, 0.0957) | 0.0635  (0.0464, 0.0869) |
