# Supplementary material for: Type of Track and Trigger system and incidence of in-hospital cardiac arrest: an observational registry-based study
Source: BMC Health Serv Res. 2020 Sep 18;20:885. doi: 10.1186/s12913-020-05721-5 (PMC7501601; doi:10.1186/s12913-020-05721-5)
Supplement: Supplementary file 4 — Additional file 4 : Table S4. Impact of interventions on IHCAs after restriction to hospitals reporting a change in intervention. [file 12913_2020_5721_MOESM4_ESM.doc]

***Supplementary Table S4*** Impact of interventions on IHCAs after restriction to hospitals reporting a change in intervention

|  | **Case mix adjusted incidence rate ratio (95% CI)** | |
| --- | --- | --- |
| **Track and trigger** | Individual intervention | Combined interventions |
| N=52 hospitals changing from non-NEWS to NEWS | | |
| Non-NEWS | reference | reference |
| NEWS | 0.946 (0.898, 0.995) | 0.948 (0.900, 0.999) |
| p-value for difference in levels | P=0.033 | P=0.045 |
| N=18 hospitals changing from paper to electronic TTS | | |
|  |  |  |
| Paper | reference | Reference |
| Electronic | 0.934 (0.857, 1.016) | 0.937 (0.859, 1.022) |
| p-value for difference in levels | P=0.113 | P=0.141 |
