# Supplementary material for: Type of Track and Trigger system and incidence of in-hospital cardiac arrest: an observational registry-based study
Source: BMC Health Serv Res. 2020 Sep 18;20:885. doi: 10.1186/s12913-020-05721-5 (PMC7501601; doi:10.1186/s12913-020-05721-5)
Supplement: Supplementary file 5 — Additional file 5 : Table S5a. Full model results (presented as odds ratios and 95% confidence intervals) for models of the association between TTS interventions and 30-day survival following IHCA. Table S5b. Full model results (presented as incidence rate ratios and 95% confidence intervals) for models of the association between TTS interventions and hospital survival in all admissions. [file 12913_2020_5721_MOESM5_ESM.docx]

***Supplementary Table S5a Full model results (presented as odds ratios and 95% confidence intervals) for models of the association between TTS interventions and 30-day survival following IHCA***

| **Variables** | **Model 8** |
| --- | --- |
| **Fixed effects parameters** |  |
| NEWS/NEWS-based (vs non-NEWS) | 1.105 (0.946, 1.292) |
| Electronic (vs paper) | 1.150 (0.922, 1.435) |
| Age (vs <65 years) |  |
| 65-74 years | 0.595 (0.519, 0.683) |
| 75-84 years | 0.428 (0.377, 0.487) |
| 85+ years | 0.290 (0.251, 0.337) |
| Female (vs male) | 1.110 (1.007, 1.223) |
| Ethnicity (vs white) |  |
| Asian/Asian British | 1.322 (1.041, 1.680) |
| Black/Black British | 1.382 (0.979, 1.951) |
| Any other ethnic group | 0.784 (0.480, 1.280) |
| Not stated or missing | 0.777 (0.633, 0.955) |
| Decile of Index of Multiple Deprivation (vs least deprived 10%) |  |
| Less deprived 10-20% | 0.976 (0.770, 1.237) |
| Less deprived 20-30% | 1.037 (0.826, 1.301) |
| Less deprived 30-40% | 1.015 (0.811, 1.271) |
| Less deprived 40-50% | 1.065 (0.850,1.333) |
| More deprived 40-50% | 0.862 (0.685, 1.086) |
| More deprived 30-40% | 0.934 (0.746, 1.168) |
| More deprived 20-30% | 0.890 (0.708, 1.119) |
| More deprived 10-20% | 0.884 (0.702, 1.114) |
| Most deprived 10% | 1.021 (0.816, 1.278) |
| Charlson index of comorbidity (vs no comorbidity) |  |
| One comorbidity | 0.772 (0.672, 0.888) |
| Two comorbidities | 0.523 (0.448, 0.609) |
| Three or more comorbidities | 0.505 (0.427, 0.598) |
| Emergency admission | 0.502 (0.431, 0.584) |
| Cardiac comorbidity | 1.148 (1.015, 1.300) |
| Main diagnosis (vs any other) |  |
| Circulatory | 1.304 (1.163, 1.463) |
| Respiratory | 0.636 (0.548, 0.738) |
| Reason for admission to hospital (vs trauma) |  |
| Medical | 0.910 (0.716, 1.156) |
| Elective/scheduled surgery | 1.521 (1.142, 2.028) |
| Emergency/urgent surgery | 0.850 (0.639, 1.131) |
| Outpatient | 1.974 (0.447, 8.707) |
| Presenting rhythm (vs VF/VT) |  |
| Asystole | 0.068 (0.056, 0.082) |
| PEA | 0.194 (0.171, 0.221) |
| Bradycardia | 0.529 (0.278, 1.006) |
| Other or missing | 0.829 (0.717, 0.958) |
| Annual trend | 1.030 (0.984, 1.079) |
| Season (vs Jan-Mar) |  |
| Apr-Jun | 1.067 (0.933, 1.221) |
| Jul-Sep | 1.049 (0.918, 1.199) |
| Oct-Dec | 0.927 (0.814, 1.055) |
| **Random effects parameters** |  |
| Sigma_u_ | 0.287 (0.221, 0.372) |

***Supplementary Table S5b Full model results (presented as incidence rate ratios and 95% confidence intervals) for models of the association between TTS interventions and hospital survival in all admissions***

| **Variables** | **Model 9** |
| --- | --- |
| **Fixed effects parameters** |  |
| NEWS/NEWS-based (vs non-NEWS) | 0.9999 (0.9982, 1.0015) |
| Electronic (vs paper) | 1.0010 (0.9987, 1.0034) |
| Age (vs <25 years) |  |
| 25-34 years | 0.9983 (0.9959, 1.0006) |
| 35-44 years | 0.9989 (0.9964, 1.0015) |
| 45-54 years | 0.9986 (0.9959, 1.0013) |
| 55-64 years | 0.9947 (0.9920, 0.9973) |
| 65-74 years | 0.9868 (0.9842, 0.9894) |
| 75-84 years | 0.9667 (0.9642, 0.9693) |
| 85+ years | 0.9229 (0.9202, 0.9255) |
| Female (vs male) | 1.0012 (1.0000, 1.0025) |
| Ethnicity (vs white) |  |
| Asian/Asian British | 1.0044 (1.0017, 1.0072) |
| Black/Black British | 1.0041 (1.0003, 1.0079) |
| Any other ethnic group | 1.0003 (0.9963, 1.0044) |
| Not stated or missing | 0.9934 (0.9912, 0.9957) |
| Decile of Index of Multiple Deprivation (vs least deprived 10%) |  |
| Less deprived 10-20% | 0.9996 (0.9969, 1.0024) |
| Less deprived 20-30% | 0.9993 (0.9965, 1.0020) |
| Less deprived 30-40% | 0.9995 (0.9968, 1.0022) |
| Less deprived 40-50% | 0.9994 (0.9967, 1.0021) |
| More deprived 40-50% | 0.9993 (0.9966, 1.0020) |
| More deprived 30-40% | 0.9991 (0.9964, 1.0018) |
| More deprived 20-30% | 0.9990 (0.9963, 1.0017) |
| More deprived 10-20% | 0.9993 (0.9966, 1.0020) |
| Most deprived 10% | 0.9996 (0.9970, 1.0023) |
| Charlson index of comorbidity (vs no comorbidity) |  |
| One comorbidity | 0.9922 (0.9907, 0.9937) |
| Two comorbidities | 0.9683 (0.9664, 0.9702) |
| Three or more comorbidities | 0.9438 (0.9414, 0.9461) |
| Emergency admission | 0.9806 (0.9792, 0.9819) |
| Cardiac comorbidity | 0.9979 (0.9956, 1.0001) |
| Main diagnosis (vs any other) |  |
| Circulatory | 0.9778 (0.9758, 0.9799) |
| Respiratory | 0.9378 (0.9357, 0.9399) |
| Pregnancy | 0.9825 (0.9804, 0.9847) |
| Annual trend | 1.0017 (1.0012, 1.0023) |
| Season (vs Jan-Mar) |  |
| Apr-Jun | 1.0032 (1.0016, 1.0049) |
| Jul-Sep | 1.0040 (1.0024, 1.0056) |
| Oct-Dec | 1.0014 (0.9998, 1.0030) |
| **Random effects parameters** |  |
| Alpha | 2.89 x 10^-6^  (9.87 x 10^-7^, 8.43 x 10^-6^) |
